# Rapid urine lipoarabinomannan assay as a clinic-based screening test for active tuberculosis at HIV diagnosis

**DOI:** 10.1186/s12890-016-0316-z

**Published:** 2016-11-14

**Authors:** Paul K. Drain, Elena Losina, Sharon M. Coleman, Janet Giddy, Douglas Ross, Jeffrey N. Katz, Ingrid V. Bassett

**Affiliations:** 1 Department of Global Health, University of Washington, 325 Ninth Ave, UW Box 359927, Seattle, WA 98104-2420 USA; 2 Department of Medicine, University of Washington, Seattle, WA USA; 3 Department of Epidemiology, University of Washington, Seattle, WA USA; 4 Medical Practice Evaluation Center, Harvard Medical School, Boston, USA; 5 Division of Infectious Diseases, Harvard Medical School, Boston, USA; 6 Division of General Internal Medicine, Harvard Medical School, Boston, USA; 7 Department of Surgery, Massachusetts General Hospital, Harvard Medical School, Boston, USA; 8 Department of Orthopedic Surgery, Brigham and Women’s Hospital, Harvard Medical School, Boston, USA; 9 Boston University School of Public Health, Boston, USA; 10 Department of Medicine, McCord Hospital, Durban, South Africa; 11 Department of Medicine, St. Mary’s Hospital, Durban, South Africa

**Keywords:** Tuberculosis, HIV/AIDS, Lipoarabinomannan (LAM), Urine, Diagnostic screening, South Africa

## Abstract

**Background:**

World Health Organization (WHO) recommends tuberculosis (TB) screening at HIV diagnosis. We evaluated the inclusion of rapid urine lipoarabinomannan (LAM) testing in TB screening algorithms.

**Methods:**

We enrolled ART-naïve adults who screened HIV-infected in KwaZulu-Natal, assessed TB-related symptoms (cough, fever, night sweats, weight loss), and obtained sputum specimens for mycobacterial culture. Trained nurses performed clinic-based urine LAM testing using a rapid assay. We used diagnostic accuracy, negative predictive value (NPV), and negative likelihood ratio, stratified by CD4 count, to evaluate screening for culture-positive TB.

**Results:**

Among 675 HIV-infected adults with median CD4 of 213/mm^3^ (interquartile range 85-360/mm^3^), 123 (18%) had culture-confirmed pulmonary TB. The WHO-recommended algorithm of any TB-related symptom had a sensitivity of 77% [95% confidence interval (CI) 69-84%] and NPV of 89% (95% CI 84-92%) for identifying active pulmonary TB. Including the LAM assay improved sensitivity (83%; 95% CI 75-89%) and NPV (91%; 95% CI 86-94%), while decreasing the negative likelihood ratio (0.45 versus 0.57). Among participants with CD4 < 100/mm^3^, including urine LAM testing improved the negative predictive value of symptom based screening from 83% to 87%. All screening algorithms with urine LAM performed better among participants with CD4 < 100/mm^3^, compared to those with CD4 ≥ 100/mm^3^.

**Conclusion:**

Clinic-based urine LAM screening increased the sensitivity of symptom-based screening by 6% among ART-naïve HIV-infected adults in a TB-endemic setting, thereby providing marginal benefit.

## Background

Over 9 million people develop active *Mycobacterium tuberculosis* (TB) infection each year, and TB remains a leading cause of HIV-related mortality [1, 2]. In 2006, the World Health Organization (WHO) suggested systematic screening for active TB in HIV-infected adults by assessing the presence of a cough for at least two weeks [3]. In 2011, following a meta-analysis of 12 observational studies, WHO recommended systematic screening by assessing the presence of any TB-related symptom—current cough, fever, night sweats, and weight loss [4–7]. Symptom-based screening misses approximately one-quarter of active TB cases among HIV-infected adults [7], and a more recent study suggested this might be as high as 76% of active TB cases in western South Africa [8]. The Xpert^®^ MTB/RIF assay has been endorsed by the WHO as the primary diagnostic test for HIV-associated pulmonary TB among those with symptoms, but is not recommended for use as a screening test. [9] A better screening strategy for HIV-associated TB is needed to accurately initiate anti-TB treatment for active disease or isoniazid preventive therapy for latent infections [10].

A rapid urine lateral flow assay to detect lipoarabinomannan (LAM), a glycolipid released from the cell wall of TB, has been shown to reduce mortality among HIV-infected hospitalized patients with TB-related symptoms when used to guide anti-TB treatment [11, 12]. In two clinic-based studies, we demonstrated that the rapid urine LAM assay had poor overall diagnostic sensitivity (28-41%) to be used as a stand-alone TB screening test at HIV diagnosis [13, 14]. With similar results from another outpatient screening study [15, 16], the WHO recently recommended against using the urine LAM assay as a TB screening test. [17] However, since the rapid LAM assay can be easily performed by nurses in a clinic [13, 14], we sought to determine if urine LAM testing might augment symptom-based TB screening at HIV diagnosis in a high TB-endemic region of South Africa.

## Methods

### Study design and participant selection

We conducted a prospective, clinic-based study enrolling consecutive ART-naïve HIV-infected adults in the ambulatory clinical areas of two hospitals and two municipal health centers in KwaZulu-Natal, South Africa from October 2011 to January 2014. The parent study has been described in detail [18]. Eligible participants were adults (≥18 years), not known to be pregnant, and not having received anti-TB therapy within three months. Ethics committees of the two local hospitals (McCord Hospital and St. Mary’s Hospital) and Partners HealthCare in Boston [Protocol #: 2006-P-001379/40] approved the study, and all participants provided written informed consent.

### Study procedures

Prior to study commencement, a representative from Alere Inc., the manufacturer of the rapid LAM assay, conducted a training session for study nurses. The session reviewed the procedures and interpretation of the Determine™ TB LAM assay (Alere Inc., Waltham, USA), and study nurses practiced performing and interpreting the urine LAM assay until comfortable. Throughout the study, LAM tests and reagents were maintained in a sealed pouch and stored out of direct sunlight, in accordance with the manufacturer’s specifications. Regular oversight of the nurses’ LAM testing procedures ensured continued testing competence and proficiency.

At participant enrollment, trained study nurses collected demographic and clinical information, including TB-related symptoms—current cough, fever, night sweats, and weight loss. Study nurses tested urine samples for LAM using the Determine™ TB LAM assay and interpreted results after 25 minutes. Participants provided one respiratory sputum sample, and those unable to provide an expectorated sputum sample received sputum induction with 3% hypertonic saline using a nebulizer (WH-802, Yuehua Medical Instrument Factory Co.; Guangdong, China). Sputum samples were decontaminated with N-acetyl L Cysteine and NaOH to a final concentration of 1.25% before being centrifuged at 3,000 revs for 20 minutes and resuspended in 1 ml of 7H9 broth. 0.5 ml of the suspension was inoculated into Bactec™ 960 mycobacterial growth indicator tubes (MGIT™) (BD; Franklin Lakes, USA), and solid culture Middlebrook 7H11 agar medium. *M. tuberculosis* was confirmed using niacin and nitrate testing. Participants received chest radiography, as indicated, which was interpreted by a certified radiologist. All participants were offered treatment in accordance with local and South African Department of Health guidelines [19].

### Statistical analyses

Participants were considered culture-positive if *M. tuberculosis* was identified from either liquid or solid culture media, and we used culture of sputum as the accepted gold-standard diagnostic test. [20] We categorized LAM test results as positive vs. negative, using the manufacturer’s 5-grade reference card. We defined a positive LAM test as grade 1 or higher, since this study was conducted before the reference card was changed to grade 2 or higher and in order to maximize diagnostic sensitivity for screening algorithms. We calculated diagnostic sensitivity, specificity, and negative predictive values of various screening algorithms, both with and without the urine LAM assay. Combined algorithms allowed for either screening test modality to be positive, since requiring multiple modalities to be positive lowered diagnostic sensitivity to an unacceptable level (29%) for a screening algorithm. We stratified algorithms above and below CD4 100/mm^3^. We used Fisher’s exact test to compare diagnostic sensitivity, specificity, and negative predictive value between groups. We also calculated negative likelihood ratios [the probability of a person who has the disease testing negative divided by the probability of a person who does not have the disease testing negative; (1-sensitivity/specificity)] to determine whether a test result changed the probability of excluding disease. The formula for calculating negative likelihood ratio includes both sensitivity and specificity, so a reduced value (< 1) indicates a favorable balance between sensitivity and specificity for a diagnostic screening “rule out” test. The WHO considers the negative predictive value (NPV; the proportion of algorithm-negative subjects who proved NOT to have TB) to be the most important parameters for a screening algorithm to exclude active TB disease [6]. We used the likelihood ratio to generate post-test probabilities based on estimated population TB prevalences of 5%, 10% and 20%. We calculated exact 95% confidence intervals (CI), reported two-tailed *p*-values (α = 0.05), and used SAS 9.4 (Cary, USA).

## Results

We enrolled 757 ART-naïve HIV-infected adults. After excluding 31 people without a urine specimen, and 51 people who had no mycobacterial culture results, our analyses included 675 adults. Mean age was 34 years (standard deviation ±10 years), 359 (53%) participants were male, 58 (9%) participants reported a prior TB infection, 165 (24%) participants were current smokers, and 3 (< 1%) participants were taking a diuretic medication (Table 1). The median CD4 count was 213/mm^3^ (interquartile range 85-360/mm^3^).

**Table 1:** Cohort characteristics of HIV-infected adults (*N* = 675).

	Mean ± SD or *N* (%)
Demographics	
Age (years)	34 ± 10
Male gender	359 (53)
Clinical	
Prior TB infection	58 (9)
Currently Smoke	165 (24)
Taking a Diuretic Medication	3 (1)
TB-related Symptoms	
Any TB-related Symptom	425 (63)
Current cough	242 (36)
Fever	220 (33)
Night sweats	211 (31)
Weight loss	264 (39)
CD4 Cell Count (median/IQR)/mm^3^ (*N* = 611)	213 (85, 360)
≥ 100 cells/mm^3^	437 (74)
< 100 cells/mm^3^	174 (29)
Radiography	
Abnormal chest x-ray	52 (8)
Rapid Urine LAM testing	
Positive by Rapid Urine LAM Test	89 (13)
Tuberculosis test results	
Sputum smear acid-fast bacilli positive	36 (5)
Culture-confirmed pulmonary TB positive	123 (18)

*IQR* interquartile range, *LAM* lipoarabinomannan, *SD* standard deviation, *TB* tuberculosis

Overall, 425 (63%) participants reported ≥ 1 TB-related symptom, and each TB-related symptom was reported by 31-39% of participants. Fifty-two (8%) participants had an abnormal chest x-ray, 36 (5%) people were sputum smear AFB positive, and 89 (13%) participants were positive by a single urine LAM assay. 123 people had culture confirmed pulmonary TB, and the estimated prevalence of culture-confirmed pulmonary TB was 18.2% (95% CI 15-21%). Prevalence of TB was 30% (71/238) and 12% (52/437) among those with CD4 < 100/mm^3^ and CD4 ≥ 100/mm^3^, respectively.

The diagnostic sensitivity of presence of a cough alone was 54% (95% CI 44-63%) (Table 2). The WHO-recommended symptom-based screening algorithm (i.e. presence of any TB-related symptom) had a diagnostic sensitivity of 77% (95% CI 69-84%) and specificity of 40% (95% CI 36-44%). The combination of any TB-related symptom or abnormal chest radiography had 83% (95% CI 75-89%) sensitivity. The negative predictive value was 89% (95% CI 84-92%) for symptom-based screening, and 91% (95% CI 87-95%) when including chest radiography.

**Table 2:** Diagnostic accuracy of symptoms and rapid testing for diagnosing culture-confirmed pulmonary tuberculosis.

	Sensitivity		Specificity		Positive PV	Negative PV
	Positive/Total	% (95% CI)	Negative/Total	% (95% CI)	% (95% CI)	% (95% CI)
Existing screening algorithms						
Cough	66/123	54 (44–63)	376/552	68 (64–72)	27 (22–33)	87 (83–90)
Any TB-related Symptom^a^	95/123	77 (69–84)	222/552	40 (36–44)	22 (18–27)	89 (84–92)
Any TB-related Symptom^a^ or Abnormal chest x-ray	102/123	83 (75–89)	219/552	40 (36–44)	23 (20–28)	91 (87–95)
Urine LAM alone						
Urine LAM Test Positive	38/123	31 (23–39)	508/552	92 (89–94)	46 (35–58)	86 (83–88)
Adding urine LAM to existing screening algorithms						
Cough or Urine LAM Positive	84/123	68 (59–76)	353/552	63 (60–68)	30 (24–35)	90 (87–93)
Any TB-related Symptom^a^ or Urine LAM Positive	102/123	83 (75–89)	205/552	37 (33–41)	23 (19–27)	91 (86–94)
Any TB-related Symptom^a^ or Abnormal chest x-ray or Urine LAM Positive	107/123	87 (80–92)	208/552	38 (34–42)	24 (20–28)	93 (89–96)

*CI* confidence interval, *LAM* lipoarabinomannan, *PV* predictive value, *TB* tuberculosis

^a^ TB-related symptoms were current cough, fever, night sweats, and weight loss

The urine LAM assay identified 25% (7/28) of TB-infected adults who did not have any TB-related symptom. The diagnostic sensitivity when incorporating the urine LAM assay with symptom-based screening was 83% (95% CI 75-89%), and with symptom-based screening plus chest radiography was 87% (95% CI 80-92%). Diagnostic specificity decreased by 2-3% in both algorithms, and was 95% among participants without TB-related symptoms. The addition of the urine LAM assay increased the negative predictive value by 2% in both screening algorithms. The highest negative predictive values were any TB-related symptom or urine LAM positive (91%; 95% CI 86-94%), and a combination of symptom screening, urine LAM, and chest radiography (93%; 95% CI 89-96%).

All screening algorithms, except those with current cough as the only TB-symptom, had higher diagnostic sensitivity among participants with CD4 < 100/mm^3^, as compared to those with CD4 ≥ 100/mm^3^ (Table 3). These differences were significant for symptom-based screening (*p* = 0.05) and symptoms plus chest radiography (*p* = 0.03). All algorithms had significantly lower diagnostic specificities among participants with CD4 < 100/mm^3^ (*p*-values <0.001). A combination of symptom screening, urine LAM, and chest radiography had a negative predictive value of 94% (95% CI 89-97%) among participants with CD4 ≥ 100/mm^3^, and 91% (95% CI 80-97%) among participants with CD4 < 100/mm^3^.

**Table 3:** Diagnostic accuracy of TB-related symptoms with and without urine LAM testing for diagnosing culture-confirmed pulmonary tuberculosis by CD4 count ≥ and <100 cell/mm^3^

	Sensitivity			Specificity			Negative PV	
	Positive/Total	% (95% CI)	*p*-value	Negative/Total	% (95% CI)	*p*-value	% (95% CI)	*p*-value
Cough			0.35			0.005		<0.0001
CD4 ≥ 100 cells/mm^3^	25/52	48 (34–62)		270/385	70 (65–75)	---	91 (87–94)	---
CD4 < 100 cells/mm^3^	41/71	58 (45–69)		106/167	63 (56–71)	---	78 (70–85)	---
Cough or Urine LAM Positive			0.24			0.007		0.0002
CD4 ≥ 100 cells/mm^3^	32/52	62 (47–75)	---	255/385	66 (61–71)	---	93 (90–96)	---
CD4 < 100 cells/mm^3^	52/71	73 (61–83)	---	98/167	59 (51–66)	---	84 (76–90)	---
Any TB-related Symptom^a^			0.05			<0.0001		0.0007
CD4 ≥ 100 cells/mm^3^	35/52	67 (53–80)	---	168/385	44 (39–49)	---	91 (86–95)	---
CD4 < 100 cells/mm^3^	60/71	85 (74–92)	---	54/167	32 (25–40)	---	83 (72–91)	---
Any TB-related Symptom^a^ or Urine LAM Positive			0.09			<0.0001		0.004
CD4 ≥ 100 cells/mm^3^	39/52	75 (61–86)	---	159/385	41 (36–46)	---	92 (87–96)	---
CD4 < 100 cells/mm^3^	63/71	89 (79–95)	---	52/167	31 (24–39)	---	87 (75–94)	---
Any TB-related Symptom^a^ or Abnormal chest x-ray			0.03			<0.0001		0.007
CD4 ≥ 100 cells/mm^3^	38/52	73 (59–84)	---	168/385	44 (39–49)	---	92 (88–96)	---
CD4 < 100 cells/mm^3^	64/71	90 (81–96)	---	51/167	31 (24–38)	---	88 (77–95)	---
Any TB-related Symptom^a^ or Abnormal chest x-ray or Urine LAM Positive			0.06			<0.0001		0.03
CD4 ≥ 100 cells/mm^3^	41/52	79 (65–89)	---	159/385	41 (36–46)	---	94 (89–97)	---
CD4 < 100 cells/mm^3^	66/71	93 (84–98)	---	49/167	29 (23–37)	---	91 (80–97)	---

*CI* confidence interval, *LAM* lipoarabinomannan, *TB* tuberculosis

^a^TB-related symptoms were current cough, fever, night sweats, and weight loss

We calculated negative likelihood ratios, which include sensitivity and specificity in a single formula, to identify the algorithm with lowest and therefore most favorable screening profile (Table 4). Overall, presence of any TB-related symptom had a negative likelihood ratio of 0.57 (95% CI 0.40-0.80), which reduces the post-test probability of a patient having active TB from 18% to 11%. The combination of symptom-based screening and chest radiography had a negative likelihood ratio of 0.43 (95% CI 0.29-0.64), which was not significantly better than symptom-based screening alone. Incorporating the urine LAM assay into these screening algorithms lowered the negative likelihood ratios to 0.45 (95% CI 0.30-0.68) and 0.34 (95% CI 0.22-0.55), respectively. Including urine LAM in the symptom-based screening algorithm lowered the probability of active TB from 11% (using symptom-based screening alone) to 9%. All negative likelihood ratios were more favorable for participants with CD4 < 100/mm^3^, compared to those with CD4 ≥ 100/mm^3^.

**Table 4:** Screening strategies for diagnosing culture-confirmed pulmonary tuberculosis

	Screening without Urine LAM	Screening with Urine LAM	Difference
	LR- (95% CI)	LR- (95% CI)	
Entire Cohort			
Cough	0.68 (0.56–0.83)	0.50 (0.38–0.65)	−0.18
Any TB-related Symptom^a^	0.57 (0.40–0.80)	0.45 (0.30–0.68)	−0.12
Any TB-related Symptom^a^ or Abnormal chest x-ray	0.43 (0.29–0.64)	0.34 (0.22–0.55)	−0.09
Cohort with CD4 ≥ 100 cells/mm^3^			
Cough	0.74 (0.57–0.97)	0.58 (0.41–0.83)	−0.16
Any TB-related Symptom^a^	0.75 (0.50–1.12)	0.61 (0.37–0.98)	−0.14
Any TB-related Symptom^a^ or Abnormal chest x-ray	0.62 (0.39–0.98)	0.51 (0.30–0.88)	−0.11
Cohort with CD4 < 100 cells/mm^3^			
Cough	0.66 (0.50–0.89)	0.46 (0.30–0.68)	−0.20
Any TB-related Symptom^a^	0.48 (0.27–0.86)	0.36 (0.18–0.72)	−0.12
Any TB-related Symptom^a^ or Abnormal chest x-ray	0.32 (0.15–0.68)	0.24 (0.10–0.58)	−0.08

*CI* confidence interval, *LAM* lipoarabinomannan, *LR* likelihood ratio, *TB* tuberculosis

^a^TB-related symptoms were current cough, fever, night sweats, and weight loss

**Fig. 1:**
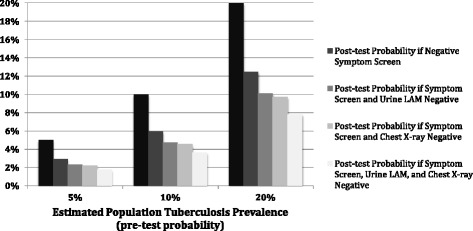
Pre- and post-test probabilities for various screening algorithms for HIV-infected populations with TB prevalence of 5%, 10%, and 20%

All clinical screening algorithms improved the ability to exclude pulmonary TB, but those benefits varied by the estimated population TB prevalence (Fig. 1). Assuming a population TB prevalence of 20%, screening negative for any TB-related symptom decreased the post-test probability of active TB disease by 37.6%. In comparison, including urine LAM in the symptom screening algorithm decreased the post-test probability by 49.4%, and including both urine LAM and chest radiography decreased the post-test probability by 60.8%. These differences were more apparent among populations with a high TB prevalence (20%) as compared to populations with a low (≤ 5%) or moderate TB prevalence (10%).

## Discussion

In this high TB-burden region of South Africa, the current symptom-based TB screening algorithm performed at initial HIV diagnosis missed nearly one-quarter of active TB cases, and had many false positive results. In particular, the WHO’s recommended symptom-based screening algorithm had a sub-optimal negative predictive value for detecting active HIV-associated TB among patients with CD4 < 100 cells/mm^3^ in this setting. Additional screening with the urine LAM assay at HIV diagnosis had marginal benefits to the existing symptom-based screening algorithm. Urine LAM testing identified one-quarter (7/28) of TB-infected adults who did not endorse any TB-related symptom, which increased the overall diagnostic sensitivity by 6%. This combined screening algorithm still missed 17% of active TB cases (83% sensitivity) among all patients, which does not meet the minimum requirements established by the WHO [21, 22]. In addition, this combination had a high rate of false positive screening results, due mostly to symptom-based screening. The overall utility of urine LAM screening improved the negative likelihood ratios, but the changes were modest. Any benefit of urine LAM appeared to be greatest in populations with a high estimated TB prevalence and CD4 < 100/mm^3^.

Our findings are consistent with a meta-analysis of 12 clinical studies that reported a similar diagnostic sensitivity (79%), but higher diagnostic specificity (50%), of symptom-based screening for culture-confirmed active TB among HIV-infected adults [7]. The meta-analysis included observational studies that collected sputum regardless of symptoms, assessed TB-related symptoms and HIV status, and used mycobacterial culture as the gold standard diagnostic test. The addition of chest radiography in the meta-analysis (defined as positive symptoms OR chest radiography) increased sensitivity to 90%, which was higher than our cohort (83%), but decreased specificity to 39%. Taken together, their calculated negative likelihood ratio was 0.43 by symptom screening alone and 0.26 when including chest radiography, which was lower (better) than our findings. However, only 3 studies included in the meta-analysis were conducted in a setting with a TB prevalence >10%—two were in Southeast Asia [23, 24], and one South African study had a small sample size with 58 culture-positive TB participants [25]. Our results further highlight the urgent need for a better TB screening algorithm among HIV-infected adults.

The negative predictive value, which changes a pre-test probability of disease into a post-test probability of disease, is a critical clinical measure for excluding TB disease by a screening algorithm [6]. When screening a population with a high estimated TB prevalence (20%), the meta-analysis reported a negative predictive value of the WHO algorithm at 90%, similar to our cohort (89%). While our cohort had a more modest increase with the addition of chest radiography (89% to 91%), the most significant impact was achieved when adding both urine LAM and chest radiography (93%). In this algorithm, 7% of those who screening negative will still have active TB disease, which highlights the continued need for an improved screening algorithm.

The consequences of inaccurate TB screening among HIV-infected adults are severe, and include missed diagnosis and inappropriate treatment of active TB, unnecessary delays for antiretroviral therapy and isoniazid preventive therapy (IPT) initiation while awaiting sputum-based diagnostic testing, inadequate provision of IPT, overprescribing of empiric TB therapy, and high loss to follow-up rates with increased program costs [8, 26]. For these reasons, the WHO has prioritized a rapid screening test that can be used by first-contact health care providers to screen for active TB, which will “triage” patients to either receive immediate IPT or diagnostic sputum-based testing with the Xpert MTB/RIF assay [21, 22]. Since the urine LAM test meets several of the WHO requirements (specificity > 70%, point-of-care test with minimal steps, time to result < 30 minutes) [21, 22], but falls short of having > 90% diagnostic sensitivity [13–15], groups are working to develop a more sensitive urinary LAM assay. In an Ethiopian cohort, increasing the LAM assay detection 50-fold by using high-avidity monoclonal antibodies improved the diagnostic sensitivity of urine LAM testing to 93% [27].

Our study had several additional strengths and limitations. Primary strengths were evaluating the urine LAM assay when used by trained nurses in ambulatory settings, which is similar to a real-world application, assessing both liquid and solid mycobacterial culture as the gold standard, enrolling people prior to HIV diagnosis and ART initiation, and assessing urine LAM among people with a range of CD4 counts. Limitations included obtaining a single sputum sample for mycobacterial culture and not performing a comprehensive evaluation for extrapulmonary TB, both of which could have misclassified people as not having TB due to an imperfect reference standard. We excluded the patients who did not provide a urine or sputum sample. Although this study was among the largest TB screening studies in HIV-infected adults [7], a limited sample size prevented the detection of statistically significant differences in improved diagnostic sensitivity and negative predictive values, or reduced negative likelihood ratios, for new screening algorithms. The WHO guidelines recommend that HIV-infected adults be screened for TB at every visit to a health facility or contact with a health worker, which we did not evaluate in our study.

## Conclusions

In conclusion, our results demonstrate a marginal benefit from the inclusion of rapid urine LAM screening to clinical symptom screening among ART-naïve HIV-infected adults in resource-constrained TB-endemic regions. Future studies should examine the role of point-of-care testing to expedite initiation of anti-TB therapy and include a cost-effectiveness analysis for clinic-based LAM screening. The current urine LAM assay is imperfect and does not solve the public health challenge of screening or diagnosing TB. However, since the principles behind the urine LAM assay are sound, an improved, next-generation urinary LAM assay could meet the criteria for a rapid, clinic-based TB screening test among HIV-infected adults in TB-endemic settings.

## Abbreviations

ART: Antiretroviral therapy
CI: Confidence interval
IPT: Isoniazid preventive therapy
IQR: Interquartile range
LAM: Lipoarabinomannan
LR: Likelihood ratio
NPV: Negative predictive value
SD: Standard deviation
TB: Tuberculosis
WHO: World Health Organization
